# The Health-Related Quality of Life of Patients Waiting for Anterior Cruciate Ligament Reconstruction Is Worse Than an Age- and Sex-Matched Population: Increasing Time on Waiting List for Surgery Was Independently Associated with a Worse Quality of Life

**DOI:** 10.1155/2022/8146897

**Published:** 2022-06-24

**Authors:** Siddharth Sripada, Harrison Loader, Man Hei Marcus Kam, Arslan Khaliq Raja, Joshua Haggart, Thomas Fawcett, Cameron Peattie, Samuel Molyneux, Nicholas Clement

**Affiliations:** ^1^University of Edinburgh Medical School, Edinburgh, UK; ^2^Edinburgh Orthopaedics, Royal Infirmary of Edinburgh, Edinburgh, UK

## Abstract

**Background:**

The aims of this study were to assess the health-related quality of life (HRQoL) of patients awaiting anterior cruciate ligament (ACL) reconstruction compared to the population at risk: whether knee-specific function was predictive of HRQoL and to identify factors associated with a worse HRQoL.

**Methods:**

Sixty-seven patients (male, *n* = 50; female, *n* = 17; mean age, 29) identified from the surgical waiting list completed a questionnaire that included demographics, BMI, time of injury, EuroQol 5-dimension (EQ-5D), Short-Form (SF-36), and International Knee Documentation Committee (IKDC) scores. Age- and sex-matched HRQoL data were obtained from population level data.

**Results:**

The mean EQ-5D score for the study cohort was significantly worse than the matched score (difference, 0.367; *p* < 0.001), and the same trend was observed for all eight dimensions of the SF-36 score. Thirty-three (49%) patients felt their health, in general, was somewhat or much worse compared to one year ago. There was a correlation between IKDC and EQ-5D scores (*r* = 0.540, *p* < 0.001). Linear regression was used to formulate the EQ-5D score: EQ-5D = (IKDCx0.013)–0.015 (constant). The SF-36 physical component and length of time on the waiting list were independently associated with the HRQoL, with each 14-point drop or for every 200 days, a clinically significant deterioration in patients' HRQoL occurred, respectively.

**Conclusions:**

Patients had a significantly worse HRQoL when compared to the age- and sex-matched population, which deteriorates with worsening physical function and increasing length of time on the waiting list. The knee-specific IKDC correlated with HRQoL and could be used to estimate the EQ-5D score.

## 1. Introduction

Anterior cruciate ligament (ACL) reconstruction is a commonly performed procedure and is associated with good functional outcomes [1]. The rate of ACL reconstruction has increased twelve-fold over the last two decades [2]. There is now a growing body of evidence that early surgical reconstruction of the ACL results in better functional outcomes compared to those patients who undergo initial nonoperative management at 2 years [3, 4]. Furthermore, ACL reconstruction aids return to sport, work, and activities of daily living and in the longer term may prevent secondary degeneration and arthritis which is associated with physical disability in younger patients [5]. Return to sports activity in amateur sports participants is only 48% eight months following reconstruction with a significant reduction in competitive level and physical commitment [6]. However, this may be related to the limited information provided by the medical and rehabilitation teams in regard to the timing of return to sport [6].

It is recognised that increasing waiting time for ACL reconstruction is associated with loss of occupation, physical deterioration, and depression due to not being able to compete in sporting activities [7]. COVID-19 has disrupted both unplanned trauma and elective/planned surgical waiting lists in most countries, with an estimated 5 years–10 years before pre-COVID-19 waiting times are restored [8, 9]. The health-related quality of life (HRQoL) of patients awaiting hip or knee arthroplasty has deteriorated during the COVID-19 pandemic due to the increased waiting times to surgery [10]. The HRQoL of younger patients awaiting ACL reconstruction is not well understood, with only a single study assessing the effect of waiting time on markers of quality of life using a nonvalidated questionnaire [7]. Furthermore, whether their HRQoL is different from that expected according to their age and sex is not known. Patients awaiting ACL reconstruction, similar to all patients awaiting surgery, will likely experience anxiety, depression, and poor quality of life, which deteriorates with increasing waiting time [11]. To the authors' knowledge, factors associated with developing a poorer HRQoL while on the waiting list have not been identified, which could be used to identify patients “at risk” and therefore prioritised for surgery. In addition, it is not clear whether knee-specific function relates to HRQoL in patients awaiting ACL reconstruction [12], which could also be used as a tool to assess the quality of life and prioritise patients.

The primary aim of this study was to assess the HRQoL of patients waiting for an ACL reconstruction compared to the population at risk. The secondary aims were to assess (1) whether knee-specific function (related to ACL activity) is associated with and predictive of HRQoL and (2) to identify patients' factors associated with a worse HRQoL while awaiting ACL reconstruction.

## 2. Methods

A cross-sectional study was undertaken. There were 101 patients identified from the orthopaedic waiting list department at the study centre waiting for an ACL reconstruction. This was a quality improvement project and was registered as such at the study centre. Therefore, no prior ethics committee approval was required. Verbal informed consent to participate was taken when patients were undertaking the questionnaire. Patients were contacted during March 2021. Patients were included if they had a symptomatic ACL injury that had either failed nonoperative management or after discussion with the patient, they felt they wanted to go forward with reconstruction primarily. Patients were excluded if they had undergone surgery by the time they were contacted (*n* = 20) or had a multiligamentous knee injury (*n* = 2). There were 67 patients that were contactable and were willing to complete the questionnaire, with 12 patients being lost to assessment. The study group (*n* = 67) consisted of 50 male patients and 17 female patients, with a combined mean age of 29 (standard deviation (SD) of 9.4, range 14–56) years. The mean body mass index preoperatively was 27.5 (SD 6.0) kg/m^2^. The mean time since injury was 637 days (SD 302), and the mean time on the waiting list was 250 days (SD 144). Patients were asked to complete a questionnaire that included patient demographics, body mass index, time of injury, EQ-5D, SF-36, and the International Knee Documentation Committee (IKDC) subjective knee evaluation form as the knee-specific measure.

The EQ-5D 5L [13] was used as the primary outcome assess to measure HRQoL. The EQ-5D general health questionnaire evaluates five domains (5D). The EQ-5D assesses mobility, self-care, usual activities, pain/discomfort, and anxiety/depression [13]. The 5L version of the EuroQoL questionnaire was used, with the responses to the five domains being recorded on five levels of severity. This index is on a scale of −0.594 to 1, where 1 represents perfect health and a score less than zero represents a health state worse than death [14]. The minimal clinically important difference (MCID) was defined as 0.08 for the EQ-5D [15]. The SF-36v2™ (QualityMetric Incorporated^©^) was also used for HRQoL as a secondary outcome measure [16, 17]. The SF-36 assesses eight dimensions that include physical function, role limitations due to physical health problems, bodily pain, vitality, social functioning, role limitations due to emotional problems, mental health, and general health. Scores for each of the dimensions range from 0 (worst level of functioning) to 100 (best level of functioning). Age and sex match population normal EQ-5D [18] and SF-36 [19] were assigned to each patient.

The IKDC score was used as the knee-specific outcome measure [20]. The IKDC is a subjective tool and contains three sections relating to knee symptoms (7 items), function (2 items), and sports activities (2 items). The overall scores range from 0 points (lowest level of function or highest level of symptoms) to 100 points (highest level of function and lowest level of symptoms).

### 2.1. Statistical Analysis

Statistical Package for Social Sciences version 17.0 (SPSS Inc., Chicago, IL, USA) was used for all data analyses. Data were assessed for normality and parametric tests where appropriate. Scalar variables were assessed using either unpaired or paired Student's *t*-test. Pearson's correlation was used to assess the relationship between linear variables. Multivariate linear regression analysis was used to identify independent predictors associated with HRQoL according to the EQ-5D score (primary outcome measure). Simple linear regression analysis was used to predict HRQoL (EQ-5D) from a patient's knee-specific score (IKDC). Bland and Altman's limits of agreement were calculated and plotted for actual and predicted EQ-5D scores. Bland and Altman recommend that the differences between each of the two measures be compared, plotting the differences against the means of the scores. If no linear relationship is observed on the Bland and Altman plot, this indicates that the statistical variation is similar. Significance was set as a *p* value of <0.05.

A power calculation was based on the primary outcome of the EQ-5D, using an effect size of 0.5 (medium), and an alpha of 5% using two-tailed analysis determined a minimum of 64 patients would be required to achieve 80% power.

There was no additional patient contact, and as such, this project was performed as a service evaluation without the need for formal ethical approval. The project was registered with the institution's audit department and was conducted in accordance with the Declaration of Helsinki and the guidelines for good clinical practice.

## 3. Results

The mean EQ-5D score was 0.557 (SD 0.272) and ranged from −0.271 to 1, which was significantly (*p* < 0.001) worse than the age- and sex-matched score (Table 1), which was greater than the MCID. The same trend was observed for all eight dimensions of the SF-36 score, with significantly (*p* < 0.001) worse scores being observed in those awaiting an ACL reconstruction (Table 1). Furthermore, 33 (49%) patients felt their health in general was somewhat or much worse when compared to one year ago.

**Figure 1:**
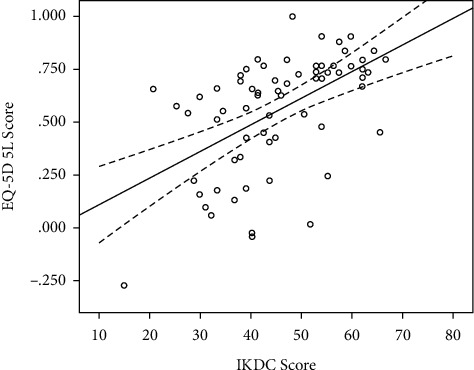
Scatter plot of IKDC score and EQ-5D with the linear line of best fit (solid black line) and the 95% confidence intervals around the mean (dashed lines).

There was a significant correlation between the knee-specific IKDC score and the EQ-5D scores (*r* = 0.540, *p* < 0.001), with a worse knee-specific function being associated with a worse HRQoL according to the EQ-5D score (Figure 1). Simple linear regression (*R*^2^ = 0.29) was used to formulate the conversion of the IKDC into the EQ-5D score:

(1) $$\begin{array}{c} \text{EQ}-5\text{D}5\text{L}=\left(\text{IKDC score}\times 0.013\right)-0.015\left(\text{constant}\right). \end{array}$$

**Figure 2:**
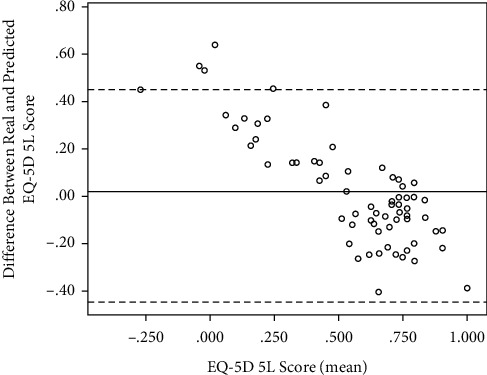
A Bland and Altman plot for the mean EQ-5D 5L score and the difference between actual and predicted scores. Solid horizontal line in the mean difference and dashed lines are 1.96 *∗* standard deviation.

This formula was used to predict the EQ-5D for each patient in the study using their actual IKDC score, which was then compared to the actual score (difference 0.2, SD 0.229). A Bland and Altman plot demonstrated that all but three data points were out with the 95% confidence intervals (Figure 2). However, a linear effect was observed with those patients with a better HRQoL (EQ-5D) score having an underestimated score using the IKDC score and vice versa for those with a worse HRQoL.

**Figure 3:**
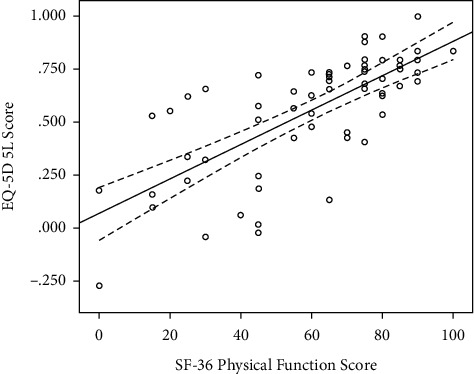
Scatter plot of physical function dimension of the SF-36 score and EQ-5D with the linear line of best fit (solid black line) and the 95% confidence intervals around the mean (dashed lines).

Increasing BMI, worse IKDC scores, and worse SF-36 scores (for all eight dimensions) were significantly associated with a worse HRQoL according to the EQ-5D score on unadjusted analysis (Table 2). However, when adjusting for confounding factors, the physical function component of the SF-36 score and the length of time on the waiting list were independently associated with the HRQoL (Table 3). A worse SF-6 physical function score was associated with a worse EQ-5D score (Figure 3), with a 14-point change being associated with a clinically significant change in the EQ-5D (MCID for EQ-5D 0.08/B from model 0.0057 (Table 3)). According to the regression model, for every 200 days (MCID for EQ-5D 0.08/B from model 0.0004 (Table 3)) spent on the waiting list for ACL reconstruction, a patient would have a clinically significant deterioration in their HRQoL.

## 4. Discussion

This study has demonstrated that the HRQoL of patients waiting for an ACL reconstruction was worse than the age- and sex-matched population, with nearly half of the patients stating their health in general was somewhat or much worse when compared to one year ago. The IKDC score correlated with HRQoL and was predictive of the EQ-5D score in patients awaiting an ACL reconstruction. A worse self-perceived SF-36 physical functional score and longer length of time on the waiting list were independently associated with a worse HRQoL, with a 14-point change or for every 200 days of wait, a patient experiences a clinically significant change in their HRQoL.

This study was carried out at a single centre, and therefore, the generalisability to a wider population may be a limitation. However, the study centre has a catchment population of nearly a million people, which may have limited any selection bias. The study surveyed predominately males (75%), and any gender-related differences in HRQoL may not have been recognised. The data collection took place during the COVID-19 pandemic, and the independent effect of this alone may have influenced the patients' HRQoL [20]. Longer waiting for surgical procedures due to COVID-19 may have increased anxiety and depression, which may explain the worse HRQoL in those awaiting an ACL reconstruction [11], which may be beyond any physical impairment from their injury. The data collected from the study population was compared to matched population data, rather than data from the same patient cohort, prior to injury. This was due to the fact that the retrospective application of the HRQoL scores used in the study was not validated.

It has been demonstrated that patients opting for early surgical intervention, rather than nonsurgical intervention, result in superior pain and quality of life scores at 2 years [3]. This suggests that delaying procedures for patients awaiting ACL reconstruction may be associated with worse postoperative outcomes, such as HRQoL, and may also increase the risk of longer-term complications such as secondary osteoarthritis of the knee [5]. There are multiple studies demonstrating that patients delaying (>6 months) ACL reconstruction had worse clinical outcomes and an increased rate of revision surgery than those patients undergoing early surgery [21]. Anstey et al. [22] and Sanders et al. [23] found early reconstruction (<6 months) reduced the risk of subsequent meniscal tears and development of arthritis in comparison to those who underwent delayed surgery. There is an association between the amount of remaining intact meniscus and the onset of arthritis in later life following ACL injury [24, 25], which may require arthroplasty surgery. Therefore, early ACL reconstruction may reduce subsequent meniscal tears as well as secondary arthritis.

Although this study was not assessing the planning or the outcome of ACL reconstruction, these should be considered. Camarda et al. [26] assessed 77 patients undergoing MRI scan assessment of the knee and assessed the association of anthropological data and tendon sizes around the knee. They found intercondylar and patella width were moderately correlated with patella tendon thickness and length and semitendinosus tendon diameter. This could be taken into account when planning surgery. The biomechanical choice of the tendon graft, potentially based on predicted tendon sizes, should also be considered. However, a recent study assessed the biomechanical properties of four different methods of suture fixation to prepare triple tendon grafts and found similar properties between the groups [27].

The HRQoL reported in the current study for patients awaiting an ACL reconstruction was significantly worse than that expected of an age- and sex-matched population. However, the HRQoL according to the EQ-5D score (0.557) may not be as limited as that observed in patients waiting for a total hip arthroplasty (THA) or total knee arthroplasty (TKA) which ranges from 0.38 to 0.57 [28]. The mean age of patients in the current study was 29 years, which is consistent with other studies awaiting ACL reconstruction [29], whereas the patients awaiting THA or TKA were older, aged between 65 and 75 years [28]. When comparing these EQ-5D scores to the age-matched scores, patients awaiting THA and TKA had an EQ-5D score between 0.3 and 0.4 points, lower than expected [18], which is similar to the current study demonstrating a 0.37 lower score for HRQoL than expected. The reason why symptomatic ACL deficiency has such an impact on HRQoL is not clear, compared to the pain and dysfunction prior to THA or TKA, but this may relate to the younger age and expectations of the patient. The younger age of the ACL group may have resulted in problems associated with return to work or even job loss and return to sport [7], which may have rippling effects on their quality of life that might not be factors in older age and retirement.

To the authors' knowledge, the current study is the first to assess the waiting time for ACL reconstruction on patients' HRQoL using validated and established patient-reported outcome measures. Salci et al. [7] assessed the association of waiting time to ACL reconstruction on markers of quality of life in a cohort of 50 patients, finding that those waiting more than 182 days for surgery were more likely to have lost or have needed to significantly modify their job. The current study supports this finding; with every 200 days on the waiting list, there was a clinically significant deterioration in the patient's HRQoL. Salci et al. [7] also demonstrated that 63% of patients felt their physical health had deteriorated and 51% felt sad/depressed most/all of the time because they could not compete in sports while awaiting surgery. The current study did not acknowledge job loss or modification, or the financial impact of this, or the influence of not returning to sporting activities, but this may explain the effect on HRQoL observed in the study cohort. It is evident from the current study and other studies that living with an ACL injury has a variety of effects on a patient's physical and mental health, which seems to continue into the postoperative and rehabilitation stages [30]. Especially given the young age of patients, their future studies, careers, and their sporting ability are at permanent risk. There is, however, evidence that ACL patients often do not regain their previous level of physical function after ACL reconstruction [31], and prolonged waiting time is an associated risk factor for this [3, 4].

The reported patient cohort demonstrated worse preoperative scores for every dimension of the SF-36 when compared to that reported by Nunez et al. [29] for their cohort of 52 patients. This variation may be because of the difference in cohort demographics, given their study was conducted in Spain, whilst the current study was conducted in the UK. However, it may also be due to a shorter waiting time from injury to surgery experienced by the patients in their study, with no patient waiting longer than 2 years. This supports the deterioration of HRQoL, according to the EQ-5D score, with a longer waiting time as observed in the current study. Furthermore, the study was conducted during the COVID-19 pandemic, which may have limited patient support due to challenges in delivering healthcare [32], and this may have impacted the HRQoL of patients on the waiting list. With waiting times inevitably prolonged due to the pandemic, it is essential to find ways to provide support to ACL patients on the waiting list and coping strategies [11].

A worse knee-specific score, according to the IKDC score, was associated with a worse HRQoL when measured using the EQ-5D score. The IKDC score measures subjective knee symptoms, function, and sports activities, which may explain the association with HRQoL. For many, an ACL injury could have been life-changing and therefore may reflect the correlation between HRQoL and knee function. Fernandez et al. [33] noted a similar relation between knee function and HRQoL, although the OKS (Oxford Knee Score) and OHS (Oxford Hip Score) were used as measures of joint-specific function in patients with osteoarthritis. They demonstrated that the OKS and OHS were useful predictors of four out of five EQ-5D dimensions, whereas the anxiety and depression dimension was not. Williams et al. [12] also concluded that there may be a considerable relationship between patients' perceived knee function and HRQoL, although their measure was specific to ACL quality of life. The current study provides a reliable conversion formula to enable conversion of the IKDC into an EQ-5D index score, which may allow comparison of HRQoL between differing patient cohorts. However, this was more reliable for EQ-5D states greater than zero.

## 5. Conclusion

In conclusion, patients awaiting ACL reconstruction had a significantly worse HRQoL when compared to age- and sex-matched population, which deteriorates with worsening perceived physical function and increasing length of time on the waiting list. The knee-specific IKDC correlated with HRQoL and could be used to estimate the EQ-5D score.

## Figures and Tables

**Table 1:** HRQoL scores of the study cohort compared to an age- and sex-matched population.

Patient-reported outcome measure	Study cohort	Matched population	Difference (95% CI)	*p* value^*∗*^
EQ-5D 5L	0.557 (0.272)	0.923	0.367 (0.300 to 0.432)	<0.001
SF-36				
Physical function	59.9 (24.2)	92.2	32.2 (26.4 to 38.1)	<0.001
Role limitations-physical	30.2 (33.6)	90.1	59.9 (51.6 to 68.1)	<0.001
Role limitations-emotional	48.7 (41.2)	84.1	35.4 (25.4 to 45.4)	<0.001
Energy/fatigue	50.0 (25.8)	63.4	13.4 (7.2 to 19.6)	<0.001
Emotional wellbeing	60.4 (18.5)	74.4	14.0 (9.5 to 18.4)	<0.001
Social functioning	56.6 (28.5)	89.7	33.1 (26.2 to 40.0)	<0.001
Pain	51.7 (27.8)	85.1	33.4 (26.7 to 40.2)	<0.001
General health	57.0 (19.5)	74.5	17.5 (12.8 to 22.2)	<0.001

^*∗*^ *t*-test.

**Table 2:** Patient variables associated with EQ-5D 5L score on unadjusted analysis for those awaiting ACL reconstruction.

Variables		Correlation coefficient	Difference	*p* value^*∗*^
Age		−0.147		0.236
Sex	Male (*n* = 50)		Reference	
	Female (*n* = 17)		0.054	0.482^*∗∗*^
BMI		−0.258		0.035
IKDC score		0.540		<0.001
Physical function		0.726		<0.001
Role limitations-physical		0.358		0.003
Role limitations-emotional		0.273		0.025
Energy/fatigue		0.387		0.001
Emotional wellbeing		0.537		<0.001
Social functioning		0.558		<0.001
Pain		0.529		<0.001
General health		0.438		<0.001
Time since injury		0.203		0.099
Time on waiting list		0.215		0.081

^*∗*^Pearson correlation unless otherwise stated; ^*∗∗*^*t*-test.

**Table 3:** Patient variables associated with the EQ-5D 5L score when adjusting for confounding using regression analysis for those awaiting ACL reconstruction (*R*^2^ = 0.66).

Variable in model		B	95% CI		*p* value
			Lower	Upper	
Age		0.0006	−0.0051	0.0064	0.829
Sex	Male	Reference			
	Female	0.0138	−0.0963	0.1239	0.802
BMI		−0.0018	−0.0099	0.0064	0.667
IKDC score		0.0028	−0.0036	0.0076	0.476
Physical function		0.0057	0.0023	0.0091	0.002
Role limitations-physical		−0.0007	−0.0026	0.0012	0.489
Role limitations-emotional		−0.0006	−0.0021	0.0009	0.452
Energy/fatigue		−0.0002	−0.0029	0.0025	0.907
Emotional wellbeing		0.0034	−0.0003	0.0070	0.068
Social functioning		0.0023	−0.0002	0.0048	0.076
Pain		0.0000	−0.0026	0.0027	0.979
General health		0.0005	−0.0025	0.0035	0.731
Time since injury		0.0000	−0.0001	0.0000	0.321
Time on waiting list		0.0004	0.0000	0.0007	0.038

## Data Availability

Raw data are available from the corresponding author upon request (Siddharth Sripada, s1904392@ed.ac.uk).

## Abbreviations

ACL: Anterior cruciate ligament
COVID-19: Coronavirus disease 2019
EQ-5D: EuroQol 5-dimension
HRQoL: Health-related quality of life
IKDC: International Knee Documentation Committee
SD: Standard deviation
SF-36: Short-Form 36
CI: Confidence interval.
